# Chemoreflex sensitization occurs in both male and female rats during recovery from acute lung injury

**DOI:** 10.3389/fphys.2024.1401774

**Published:** 2024-07-22

**Authors:** Kajal Kamra, Irving H. Zucker, Harold D. Schultz, Han-Jun Wang

**Affiliations:** ^1^ Department of Cellular and Integrative Physiology, University of Nebraska Medical Center, Omaha, NE, United States; ^2^ Department of Anesthesiology, University of Nebraska Medical Center, Omaha, NE, United States

**Keywords:** acute respiratory distress syndrome, acute lung injury, bleomycin, chemoreflex, sex-based differences

## Abstract

**Introduction:**

Sex-specific patterns in respiratory conditions, such as asthma, COPD, cystic fibrosis, obstructive sleep apnea, and idiopathic pulmonary fibrosis, have been previously documented. Animal models of acute lung injury (ALI) have offered insights into sex differences, with male mice exhibiting distinct lung edema and vascular leakage compared to female mice. Our lab has provided evidence that the chemoreflex is sensitized in male rats during the recovery from bleomycin-induced ALI, but whether sex-based chemoreflex changes occur post-ALI is not known. To bridge this gap, the current study employed the bleomycin-induced ALI animal model to investigate sex-based differences in chemoreflex activation during the recovery from ALI.

**Methods:**

ALI was induced using a single intra-tracheal instillation of bleomycin (bleo, 2.5 mg/Kg) (day 1). Resting respiratory frequency (f_R_) was measured at 1-2 days pre-bleo, day 7 (D7) post-bleo, and 1 month (1 mth) post-bleo. The chemoreflex responses to hypoxia (10% O_2_, 0% CO_2_) and normoxic-hypercapnia (21% O_2_, 5% CO_2_) were measured before bleo administration (pre-bleo) and 1 mth post-bleo using whole-body plethysmography. The apnea-hypopnea Index (AHI), post-sigh apneas, and sighs were measured at each time point.

**Results:**

There were no significant differences in resting f_R_ between male and female rats at the pre-bleo time point or in the increase in resting f_R_ at D7 post-bleo. At 1 mth post-bleo, the resting f_R_ was partially restored in both sexes but the recovery towards normal ranges of resting f_R_ was significantly lower in male rats. The AHI, post-sigh apneas, and sighs were not different between male and female rats pre-bleo and 1 mth post-bleo. However, at D7 post-bleo, the male rats exhibited a higher AHI than female rats. Both male and female rats exhibited a sensitized chemoreflex in response to hypoxia and normoxic-hypercapnia with no significant differences between sexes.

**Conclusion:**

A sex difference in resting ventilatory parameters occurs post ALI with a prolonged increase in resting f_R_ and larger AHI in male rats. On the other hand, we did not find any sex differences in the chemoreflex sensitization that occurs at 1 mth post-bleo. This work contributes to a better understanding of sex-based variations in lung disorders.

## 1 Introduction

In studies involving vertebrate animals and humans, the consideration of “sex” as a critical biological variable is essential throughout the design, analysis, and reporting of research (Lee, 2018). Sex-related differences in lung development are apparent throughout life, affected by factors like variations in lung growth and maturity, as well as the influence of sex hormones (Carey et al., 2007; Silveyra et al., 2021). Pulmonary diseases such as respiratory distress syndrome, bronchiolitis, pneumonia, chronic obstructive pulmonary disease (COPD), obstructive sleep apnea (OSA), and the SARS-CoV-2 epidemic have reported sex differences (Silveyra et al., 2021).

Acute lung injury (ALI) and its severe form, acute respiratory distress syndrome (ARDS) caused by the disruption of the normal capillary endothelial barrier leads to impacting ventilatory control (Young et al., 2019). Acute respiratory failure, responsible for 10% of ICU admissions with significant mortality and morbidity, affects approximately 200,000 new cases annually in the US alone (Mowery et al., 2020). Epidemiological research on sex differences in all-cause ARDS presents contradictory findings, with some studies indicating a substantial correlation between ARDS incidence, mortality, and sex (Moss and Mannino, 2002; Agarwal et al., 2006; Phua et al., 2009; Lemos-Filho et al., 2013; Chen et al., 2015). Structural and functional variations in lung and airway development, influenced by genetic, epigenetic, hormonal, and environmental factors, contribute to these disparities (Silveyra et al., 2021). Notably, pre-term birth in males is associated with a greater disadvantage (Bancalari and Jain, 2019). Sex hormones, particularly testosterone and estrogen, play a role in influencing immune-related cells, macrophage polarization, airway smooth muscle cells, and inflammation (Becerra-Díaz et al., 2018). Clinical studies show that male infants are more susceptible to lower respiratory tract infections, bronchiolitis, respiratory distress syndrome, and bronchopulmonary dysplasia (Liptzin et al., 2015). In contrast, male children are more prone to asthma, while women exhibit a higher exacerbation risk in chronic obstructive pulmonary disease (COPD) and a lower likelihood of obstructive sleep apnea (OSA) compared to men (Han et al., 2018). Sex differences have also been studied in different models of ALI by several groups. Most importantly, all those studies focused on lung pathology post-ALI. A potential sex difference in the neural control of breathing post-ALI has not been investigated.

Carotid bodies (CBs), peripheral chemoreceptors found at the common carotid artery bifurcation, are stimulated by hypoxemia brought on by ALI. As a basic defensive response to return blood gas concentrations to normal, these chemoreceptors detect changes in pH and blood gas levels. The enhancement of sympathetic drive by acute or chronic CB activation is a well-established phenomenon. Excessive sympathetic output can cause cardiac arrhythmias, cardio-renal syndrome, metabolic syndrome, Type 2 diabetes, and impaired cardiac activity (Mark, 1995; Marcus et al., 2014; Moreira et al., 2015; Li, 2022). Previously, our laboratory has provided evidence of increased chemosensitivity during recovery from ALI in male rats (Kamra et al., 2022). Nevertheless, there remains a knowledge gap regarding the comparisons of male *vs.* female chemoreflex changes during the recovery from ALI. The current study utilizes a bleomycin-induced ALI animal model to investigate sex-based differences in chemoreflex activation under ALI conditions.

## 2 Methods

### 2.1 Ethical approval

Animals were housed in a temperature-controlled environment (22°C–25°C) with a 12 h light-dark cycle and *ad libitum* access to food and water, by standards set by the National Institutes of Health Guidelines for the Care and Use of Laboratory Animals. All experimental protocols were approved by the Institutional Animal Care and Use Committee (IACUC) of the University of Nebraska Medical Center (protocol ID no. 17-006-03 FC).

### 2.2 Animals

**FIGURE 1:**
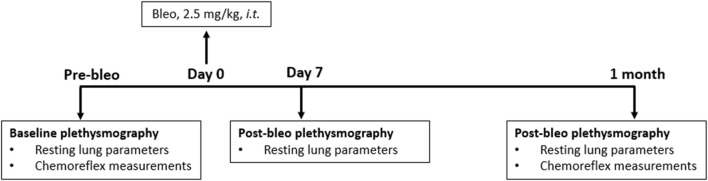
Timeline showing the experimental design.

Eighteen adult (eight male and ten female) Sprague-Dawley rats (2 - 3 months old) were used for these experiments. They were allowed to acclimate for 3 days to their new environment before the experiment. All animal experimentation (collection of ventilatory parameters during rest and hypoxic/hypercapnic gas exposure) was performed during the day (9:00–16:00 h). Delivery of bleomycin sulfate (bleo) was performed within the Department of Comparative Medicine. At the end of the experimental protocol, all animals were humanely euthanized with an overdose of pentobarbital sodium (150 mg/kg, IV). Euthanasia was confirmed by the removal of vital organs. An experimental timeline is shown in Figure 1.

### 2.3 Drugs and chemicals

Bleo was purchased from Enzo Life Sciences (New York, United States). Bleo was dissolved in saline for intra-tracheal administration. This procedure was performed within the Department of Comparative Medicine.

### 2.4 Rat model of lung injury

Rats were randomized into two experimental groups and evaluated at three time points- 1-2 days before instillation (pre-bleo), day 7 (D7) post-bleo, and 1 month (1 mth) post-bleo instillation as follows: male bleo-treated rats (n = 8) and female bleo-treated rats (n = 10). Bleo (2.5 mg/kg) was instilled on day 0 intratracheally under 2% - 3% isoflurane anesthesia.

### 2.5 Breathing and ventilatory chemoreflex function

Unrestrained whole-body plethysmography was utilized to measure ventilatory parameters-respiratory rate (f_R_), tidal volume (V_t_) and minute ventilation (V̇_E_) in conscious rats by using signals from a differential-pressure transducer (DLP 2.5, Harvard Apparatus), amplified, and connected to a PC via an acquisition system (PowerLab 35 Series) managed by LabChart (v8.1.5) software (ADInstruments, Colorado Springs, United States). Rats were acclimated to the plethysmograph chamber for 1 h each for two consecutive days before recordings. Respiratory parameters were not recorded during the acclimatization sessions. The plethysmography chambers used for this study were custom-made (Midwest Plastics Inc., Nebraska, United States) and were 10, 10.5, and 20 cm in height, width, and length, respectively. The volume channel (i.e., flow integration) was calibrated by pushing 5 mL of air using a syringe before the start of the recording. During recordings, a constant flow of gas at 3 L/min was maintained to avoid an increase in humidity, temperature, and CO_2_ levels using a manually operated flow meter (Precision Medical, Northampton, PA, United States). The body weight (in grams) of rats was recorded before each experiment. In the resting state, rats were exposed to normoxia (21% O_2_, 0% CO_2_) for baseline measurements followed by two different gas challenges-hypoxia (10% O_2_, 0% CO_2_) and normoxic hypercapnia (21% O_2_, 5% CO_2_) balanced by N_2_. The order of the gas challenge was randomized and was maintained for 5 minutes. The last 1-min-long segment without any artifacts was used for analysis. A normoxic exposure of a minimum of 10 min or more was used in between challenges. All resting ventilatory parameters considered for analysis were recorded when the rats were awake and stationary (no activity-related events were included in the analysis in the LabChart8 raw data file). V̇_E_ was calculated as the product of f_R_ and V_t_. V_t_ and V̇_E_ were normalized to body weight. All data values were extracted from LabChart8 raw data files. A 30-min recording without artifacts was recorded for all rats while they breathed room air. This was used to measure all resting ventilatory parameters and to manually extract apneas, hypopneas, sighs, and post-sigh apneas from LabChart 8 raw data files. Apneas were defined as the cessation of breathing for at least three respiratory cycles, as determined by the respiratory rate for the prior 10 s; hypopneas were defined as reductions in breath amplitude 50% of the average cycle amplitude of the preceding 10 s of regular breathing; post-sigh apneas were defined as the cessation of breathing for at least three respiratory cycles immediately after a sigh. Apneas and hypopneas were expressed as Apnea-Hypopnea Index (AHI, events/hour). Sighs and post-sigh apneas were also expressed as events/hour.

### 2.6 Statistical analysis

Data analysis in text, tables, and figures are presented as mean ± SD. Statistical evaluation was analyzed using GraphPad Prism (GraphPad Software, San Diego, CA. Version 8). For the chemoreflex comparisons, two-way ANOVA with Bonferroni corrections (male vs. Female and Pre-Bleo vs. Post-Bleo) was used with *p* ≤ 0.05 being statistically significant. Body weight and baseline respiratory parameters at multiple time points post-Bleo were compared by using two-way Repeated Measurement (RM) ANOVA with *p* ≤ 0.05 being statistically significant.

## 3 Results

### 3.1 Effect of bleomycin on body weight in male and female rats post-ALI

Body weights were measured in all rats at pre-bleo administration and post-bleo administration at D7 and 1 mth. Body weight did not change in either male or female rates during the first 7 days post-bleo (Table 1). At 1-mth post-bleo, male and female rats significantly increased body weight by 99 ± 74 g (*p* < 0.0001) and 56 ± 24 g, *p* < 0.0001), respectively (Table 1).

**TABLE 1:** Mean body weight and mean change in body weight (in grams) for male and female dose bleo rats.

	Body weight (grams)		
	W0 pre-bleo	Day 7 post-bleo	1 mth post-bleo
Male rats (n = 8)	410 ± 59	394 ± 63	510 ± 47 **
Female rats (n = 10)	187 ± 27	175 ± 17	243 ± 19 **

Values are mean ± SD; Bleo indicates bleomycin. ^**^ *p* < 0.0001 *vs.* W0 (Week 0) pre-bleo.

### 3.2 Sex-based differences in baseline respiratory parameters between male and female rats post-ALI

**FIGURE 2:**
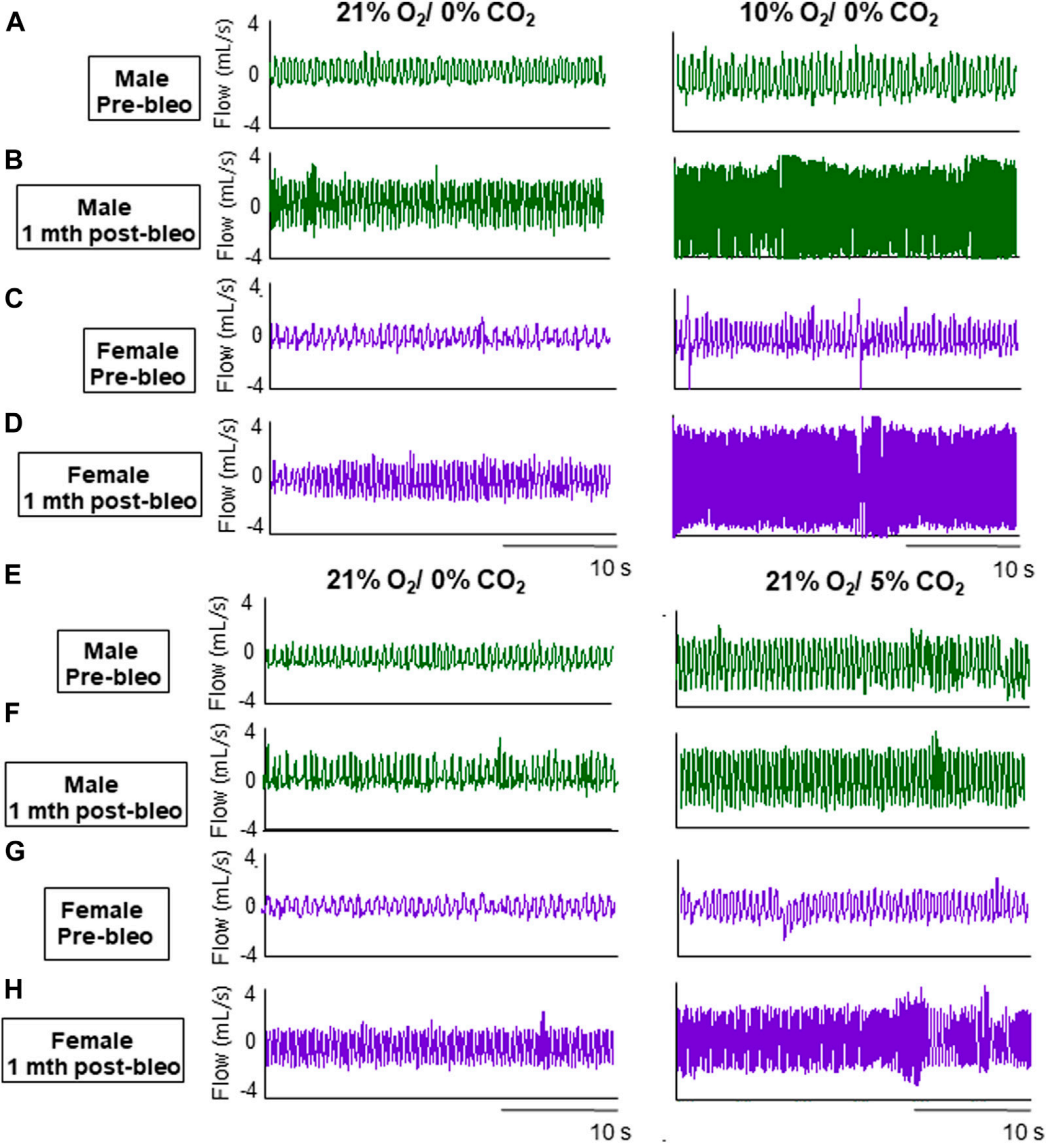
Representative ventilator tracings at normoxia, 10% hypoxia, and 5% normoxic-hypercapnia were obtained in a male and a female rat.

As noted in Figures 2A, C, E, G, male and female rats showed a steady normal resting f_R_ pattern pre-bleo with average f_R_ similar between groups (male rats = 95 ± 15 bpm; female rats = 86 ± 11 bpm (Table 2). However, as expected, consistent with our previously published data, both male (280 ± 70 bpm, *p* < 0.0001) and female (240 ± 43 bpm, *p* < 0.0001) rats exhibited a significant increase in resting f_R_ post-bleo treatment at D7 after receiving bleo compared to the pre-bleo time point (Table 2). This increase in f_R_ was partially restored at 1 mth post-bleo treatment in both groups (male = 141 ± 44 bpm (*p* = 0.0004, D7 *vs.* 1 mth post-bleo) and female = 104 ± 23 bpm (*p* < 0.0001, D7 *vs.* 1 mth post-bleo) (Table 2; Figures 2B, D, F, H). The comparison of f_R_ between male and female rats at 1 mth post-bleo time point was significantly different (*p* = 0.03) with male rats having a higher resting f_R_ than female rats (male *vs.* female: 141 ± 44 bpm *vs.* 104 ± 23 bpm). In male rats, the V_t_ and V_E_ were significant differences in D7 post-bleo when compared to pre-bleo time points. The female group also showed no significant differences in V_t,_ however, V_E_ was significantly increased at D7 post-bleo and 1 mth-post-bleo when compared to pre-bleo (*p* < 0.0001, female rats: pre-bleo *vs.* D7 post-bleo).

**TABLE 2:** Mean resting ventilatory parameters for male and female bleo rats.

		Male rats (n = 8)	Female rats (n = 10)
Resting f_R_ (BPM)	Pre-bleo	95 ± 15	86 ± 11
	D7 post-bleo	280 ± 70 ***	240 ± 43 ***
	I-mth post-bleo	141 ± 44 $ *	104 ± 23
Resting V_t_ (mL/Kg)	Pre-bleo	0.45 ± 0.15	0.63 ± 0.27
	D7 post-bleo	0.31 ± 0.1 $$ **	0.57 ± 0.18
	I-mth post-bleo	0.38 ± 0.05 $$$	0.75 ± 0.19
Resting V_E_ (mL/min/Kg)	Pre-bleo	40 ± 9.8	50 ± 27.6
	D7 post-bleo	85 ± 27 $$ **	143 ± 52 ***
	I-mth post-bleo	57 ± 20.76	74 ± 24 *

Values are mean ± SD; bleo indicates bleomycin. ^$^ (all time points *vs.* pre-bleo); * (Male *vs.* Female rats).

### 3.3 Sex-based differences in the occurrence of apneic events between male and female rats post ALI

**FIGURE 3:**
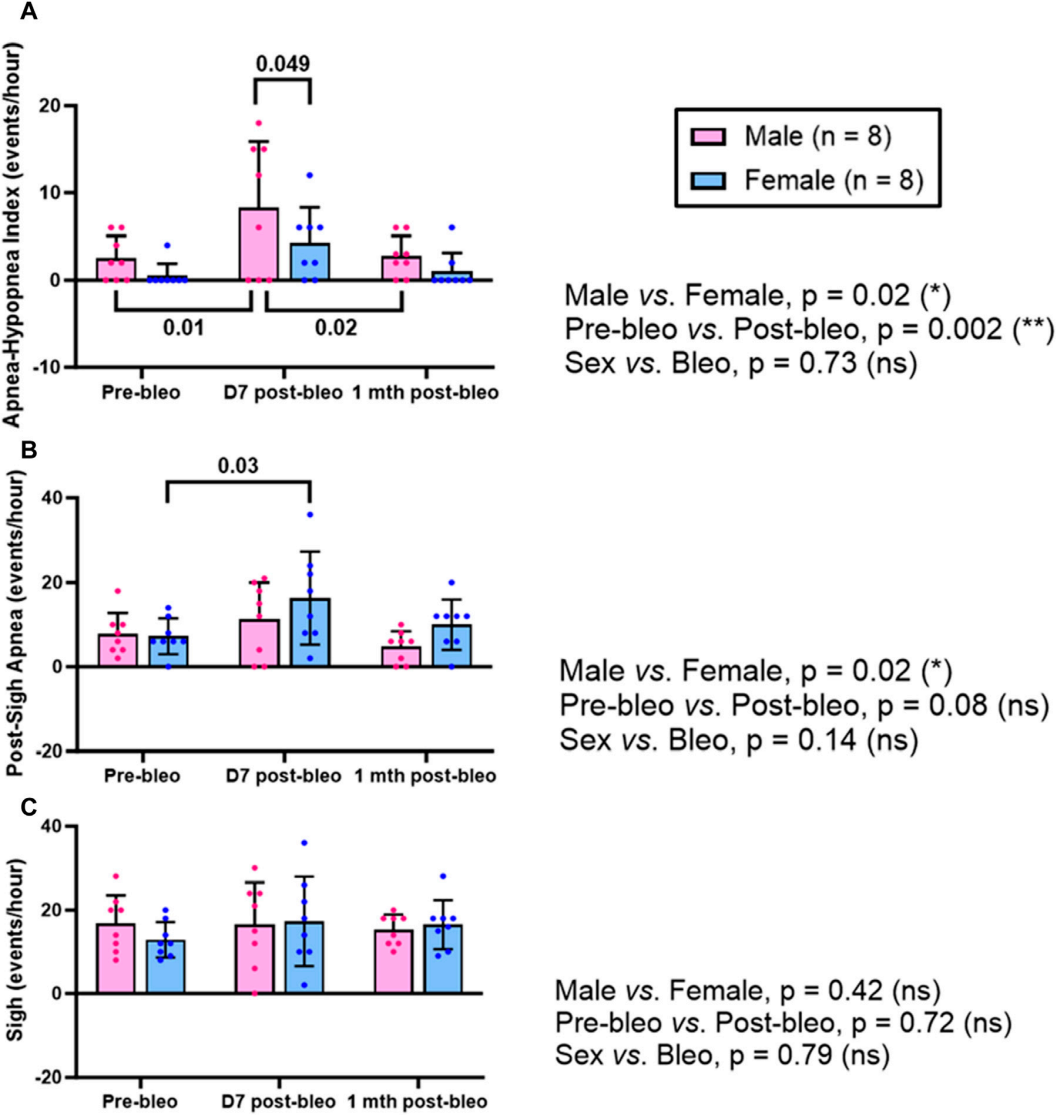
Effects of sex, bleomycin (pre-*vs.* post-bleo) and the interaction between the sex and treatment for **(A)** Apnea-hypopnea index (AHI), **(B)** Post-sigh apnea occurrence, and **(C)** Sigh occurrence in male (n = 8) and female (n = 8) rats. Two-way ANOVA, repeated measures, Values are mean ± SD.

The 30-minute-raw data recorded during normoxic gas exposure were utilized to manually extract apneas from male and female rats at three time points-pre-bleo, D7 post-bleo, and 1 mth post-bleo. Male and female rats showed a significant difference (*p* = 0.02) in the occurrence of apneas throughout the experimental timeline. The changes pre- and post-bleo were also seen to be significantly different (*p* = 0.002) (Figure 3A).

The post-sigh apneas were significantly different between male vs. female rats (*p* = 0.02). These differences however were not seen to be affected by bleo treatment (pre-vs. post-bleo, *p* = 0.08) (Figure 3B). The occurrence of sighs did not change significantly at all three time points for either sex and for pre-vs. post-bleo, revealing no sex differences for sighs during the acute and recovery phase of ALI (Figure 3C).

### 3.4 Sex-based differences in the chemoreflex sensitivity between male and female rats during the recovery from ALI

**FIGURE 4:**
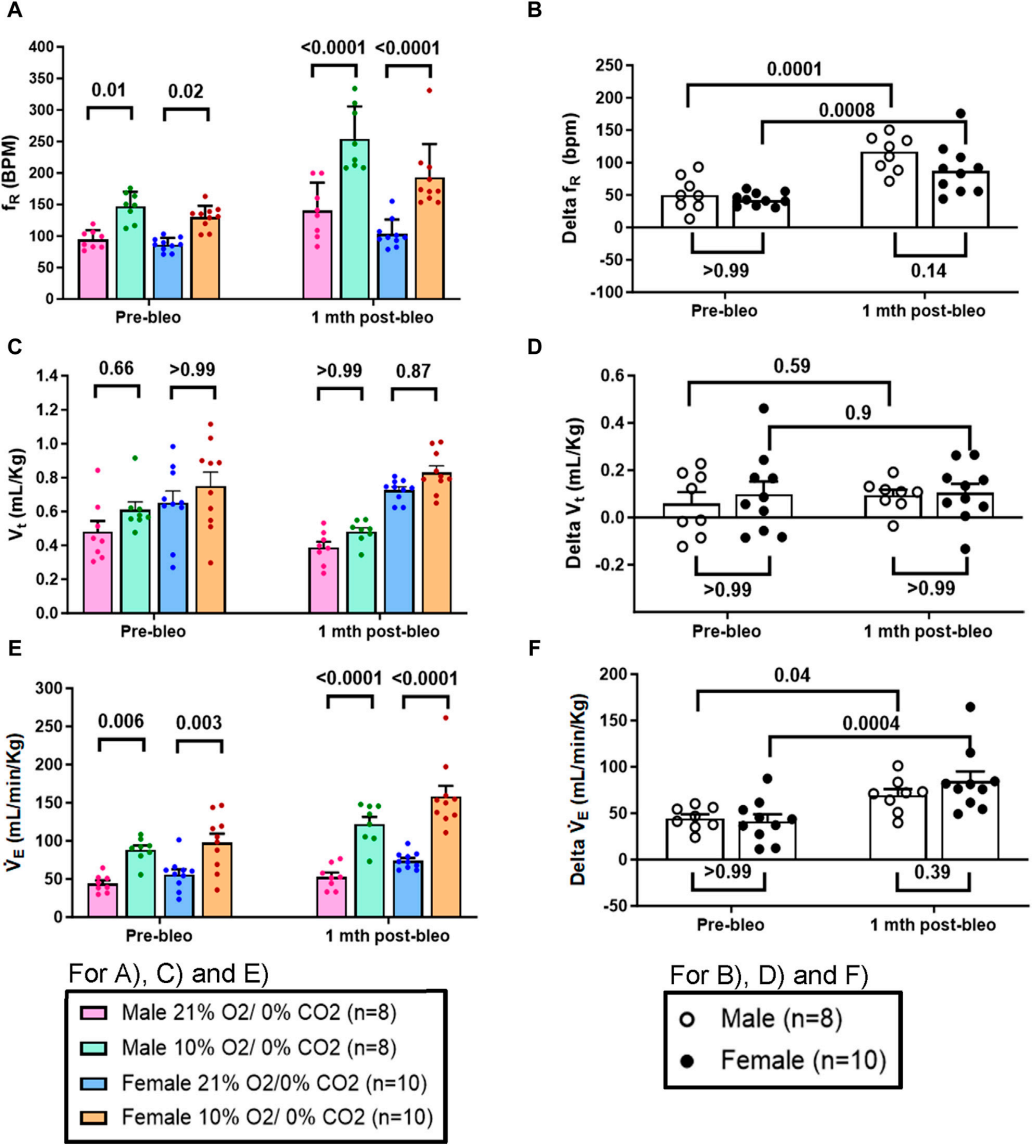
Effect of bleomycin on ventilatory parameters in male (n = 8) and female (n = 10) rats in response to chemoreflex activation with 10% O2/0% CO2. Two-way ANOVA, Values are mean ± SD: **(A)** Respiratory rate (f_R_) at normoxia *vs.* hypoxia; **(B)** Delta f_R_ (Delta is the absolute difference between the response at normoxia and hypoxia); **(C)** Tidal volume (V_t_) at normoxia *vs.* hypoxia; **(D)** Delta V_t_; **(E)** Minute ventilation (V̇_E_) at normoxia *vs.* hypoxia; **(F)** Delta V̇_E_.

The chemoreflex responses to 10% hypoxia and 5% normoxic-hypercapnia were assessed by measuring the absolute difference between 21% O_2_/0% CO_2_ and 10% O_2_/0% CO_2_ or 21% O_2_/5% CO_2_-induced responses. At pre-bleo, both male and female rats exhibited similar increases in f_R_ and V_E_ in response to 10% hypoxia (Figures 4A, C, E). At 1 mth post-bleo, there was a significant increase in chemosensitivity (Figures 4B, F) with no differences in chemoreflex activation between male *vs.* female groups of rats (Figures 4B, F). The changes in V_t_ in response to 10% hypoxia were not statistically significant for either male *vs.* female groups of rats (Figures 4C, D).

**FIGURE 5:**
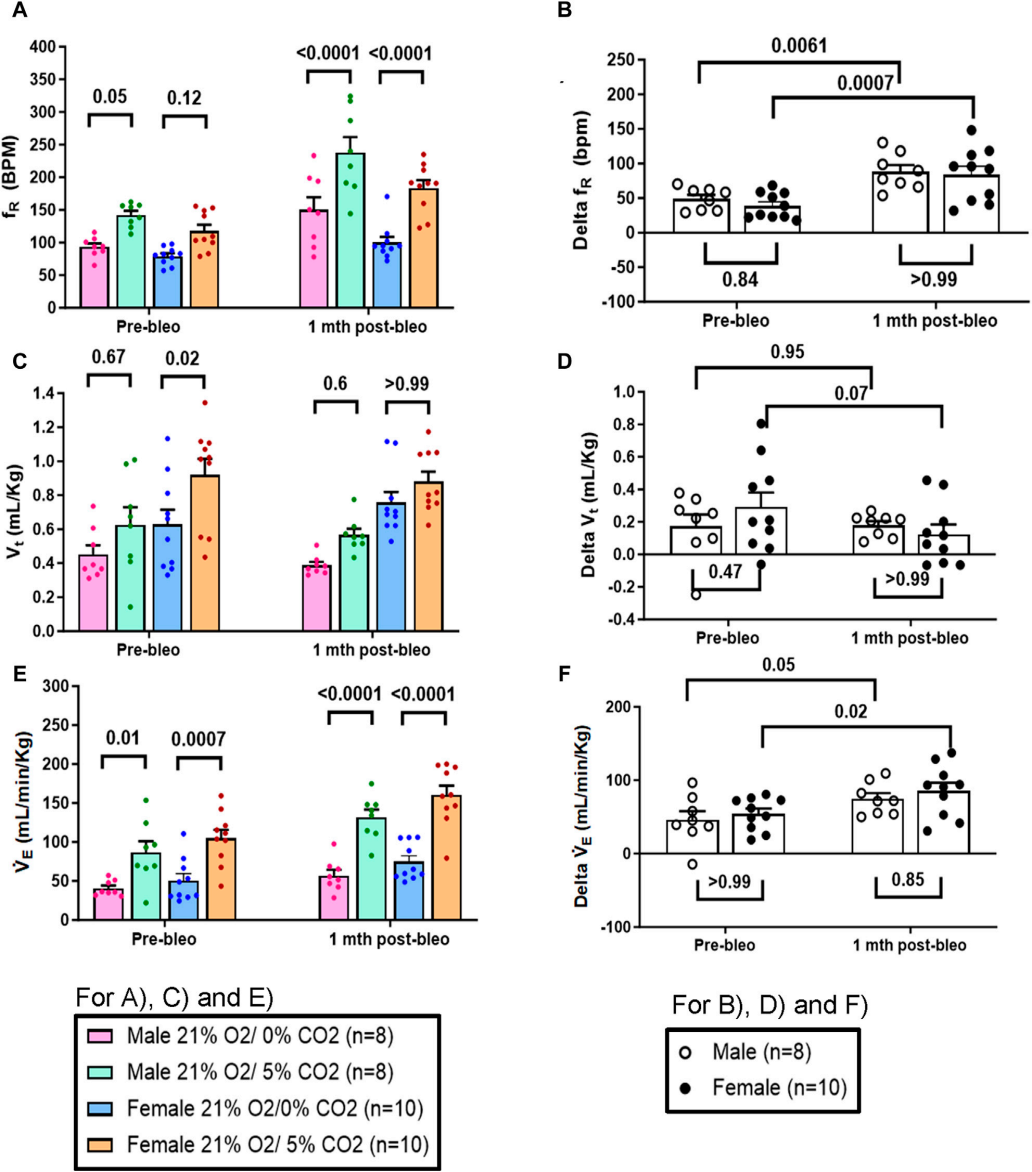
Effect of bleomycin on ventilatory parameters in male (n = 8) and female (n = 10) rats in response to 21% O2/5% CO2. Two-way ANOVA, Values are mean ± SD: **(A)** Respiratory rate (f_R_) at normoxia *vs.* normoxic-hypercapnia; **(B)** Delta f_R_ (Delta is the absolute difference between the response at normoxia and normoxic-hypercapnia); **(C)** Tidal volume (V_t_) at normoxia *vs.* normoxic-hypercapnia; **(D)** Delta V_t_; **(E)** Minute ventilation (V̇_E_) at normoxia *vs.* normoxic-hypercapnia; **(F)** Delta V̇_E_.

Similarly, in response to 5% normoxic-hypercapnia, both male and female rats showed similar changes in f_R_ and V_E_ at pre-bleo time points (Figures 5A, E). Like 10% hypoxia, both groups showed a significant increase in chemoreflex activation at 1 mth post-bleo with no difference between sexes (Figures 5B, F). The changes in V_t_ in response to 5% normoxic-hypercapnia were not statistically significant for either male *vs.* female groups of rats (Figures 5C, D).

## 4 Discussion

In this study, we examined the resting breathing parameters (f_R_, AHI, sighs, post sigh apneas) during both the acute phase (D7) and recovery phase (1 mth) of ALI, and the chemoreflex sensitization during the recovery from ALI in male and female rats. The key findings of this study are summarized as follows: 1) There were no significant differences in the resting breathing parameters (f_R_, AHI, sighs, post sigh apneas) between male and female rats. At the D7 post-bleo, resting f_R_ was significantly increased in both male and female rats but tended to increase by a larger degree in male rats. At 1 mth post-bleo, the resting f_R_ was partially restored in both sexes but the recovery towards normal ranges of resting f_R_ was significantly lower in male rats. At D7 post-bleo, the males exhibited significantly more events/hour of AHI compared to females. 2) At the pre-bleo time point, both male and female groups showed similar chemoreflex responses in f_R_ to 10% hypoxia and 5% normoxic-hypercapnia. During recovery from ALI at 1 mth post-bleo, the chemoreflex sensitivity in response to hypoxia and normoxic-hypercapnia did not exhibit sex-based differences.

Animal models, such as the two-hit model involving acid instillation followed by overventilation, have provided histological evidence of sex differences in ALI, with male mice exhibiting distinct lung edema and vascular leakage compared to female mice (Erfinanda et al., 2021). Another study observed higher levels of airway inflammation and hyperresponsiveness in male mice (Carey et al., 2007). Lingappan et al. investigated sex-specific differences in lung injury induced by hyperoxic exposure in neonate mice (Lingappan et al., 2016). They found that male neonate mice exhibited a greater cytokine response and pulmonary angiogenesis arrest compared to females (Lingappan et al., 2016). They observed similar patterns in adult male mice compared to females (Lingappan et al., 2016). On the other hand, several other studies from LPS-induced ALI models showed no significant sex difference in lung pathology. For instance, Nguyen et al. reported no differences in pulmonary injury and abnormal lung development between male and female mice exposed to early postnatal systemic LPS challenge (Nguyen et al., 2019). They also reported findings that suggest a similar innate response to early neonatal LPS exposure and the resulting pulmonary sequelae in male and female mice (Nguyen et al., 2019). Another study demonstrated no sex-based differences in LPS-induced TLR4 expression in the adult rat lung (Du et al., 2005).

A previous study from our lab using a bleo-induced ALI adult rat model demonstrated a significant increase in resting f_R_ in male ALI rats at D7 post-bleo administration (Kamra et al., 2022). The present study demonstrated no significant difference in this increase in resting f_R_ at D7 post-bleo between male and female rats. However, a trend of comparatively higher resting f_R_ was seen in adult male ALI rats at D7 post-bleo compared to female ALI rats. More importantly, the recovery from increased resting f_R_ in male rats at 1 mth post-bleo was significantly slower than in female rats. This may be due to the protective effects of female sex hormones (Speyer et al., 2005; Carey et al., 2007; Erfinanda et al., 2021). It is known that female neonate mice demonstrated a better antioxidant defense mechanism against hyperoxia-induced reactive oxygen species (ROS) (Vento et al., 2009; Tondreau et al., 2012). Estrogen’s antioxidant, vasodilatory, and anti-inflammatory properties contribute to a lower risk of cardiovascular diseases in females (Xiang et al., 2021).

In addition, we also documented a significant increase in the occurrence of apneas and hypopneas in male ALI rats at the D7 post-bleo time point. This increase was significantly increased in male rats at this time point compared to female bleo rats that also exhibited a trend towards higher apneic incidences. The post-sigh apneas were significantly higher at D7 post-bleo in female rats while the male rats exhibited a trend to show an increase. Consistent with our AHI data, clinical evidence also suggests that obstructive sleep apnea is documented to be higher in males than in females (Lin et al., 2008). In our study, we see this significant increase in apneic events at the D7 post-bleo time point as lung injury is at its peak during this period, after the first and only insult of bleo (Kamra et al., 2022). It is known that the occurrence of apneas and hypopneas are among many factors associated with increased sympathetic nerve activity, inflammation, and intermittent hypoxia, and may be caused by respiratory control instability (Javaheri et al., 2017).

Previously, we reported evidence of enhanced chemoreflex response to hypoxia and normoxic-hypercapnia in the recovery phase (1 mth post-bleo) in male ALI rats (Kamra et al., 2022). However, a potential sex difference in chemoreflex sensitivity during the recovery of ALI was not examined. The data from the present study in male and female rats corroborate the findings from our previous study in male ALI rats of chemoreflex sensitization with recovery from ALI. We now show no statistically significant differences in chemoreflex sensitization in male vs. female rats during the recovery of ALI.

Supportive evidence of no sex differences in chemosensitivity was presented by Usselman et al. who examined the effect of sex on chemoreflex regulation of muscle sympathetic nerve activity in young healthy men and women who were not using any form of hormonal contraception (Usselman et al., 2015). They observed similar sympathetic responses to chemoreflex activation in men and women (Usselman et al., 2015). Interestingly, in rats exposed to CIH, another study showed there are sex differences in respiratory-related sympathetic nerve discharge that characterize differences in the respiratory modulation of sympathetic activity after CIH (Souza et al., 2018).

Independent of sex hormones, these data in juvenile rats (Souza et al., 2018) demonstrate sexual dimorphism in the reconfiguration of respiratory and pre-sympathetic network interactions after CIH (Souza et al., 2018). It is interesting to note that in the current study, at 1 mth post-bleo, the recovery of resting f_R_ was significantly faster in female bleo rats than in male bleo rats. The generation of resting f_R_ is controlled both by intrinsic respiratory drive and thoracic neural receptors. It is possible that in the case of female bleo-rats, the female sex hormone exhibited protective effects in the reflexes controlling the resting f_R_ generation (Behan et al., 2003; Zhang et al., 2020). However, at the same time point (1 mth post-bleo), we note that the chemoreflex activation in response to both hypoxia and normoxic-hypercapnia was not different between the sexes. The peripheral and central chemoreceptors contribute to the integrated receptor input in the pons and medulla to modify the generation of rhythmic breathing during non-normoxic conditions. The effect of sex hormones on these chemoreceptors and whether estrogen has any protective effects on the respiratory rhythm generator is not well understood. It is noteworthy that sex-based differences in chemoreflex were not measured at the D7 post-bleo timepoint in the current study. According to our earlier research, male rats with moderate ALI tend to exhibit a significantly blunted chemoreflex response to hypoxia or normoxic-hypercapnia at this timepoint, whereas those with severe ALI generally have a more sensitive chemoreflex response (Kamra et al., 2022). The reasons for this disparity in chemoreflex sensitivity between male rats with moderate and severe ALI at D7—blunted versus sensitized—remain unclear. However, we observed that during the ALI recovery period, or 1-month post-ALI, both moderate and severe ALI rats developed a sensitized chemoreflex. Given that both moderate and severe ALI rats showed consistent chemoreflex sensitization at this chronic time window, we chose to focus on the recovery period in the current study due to the complex changes in chemoreflex sensitivity between these groups at the D7 post-ALI timepoint.

## 5 Conclusion

In conclusion, our study highlighted noteworthy sex differences in terms of resting f_R_, with a pronounced increase observed in males at the 1 mth post-bleo time point. Moreover, sex-specific variations were identified in acute AHI (at the D7 post-bleo), emphasizing the importance of sex as a determinant in respiratory responses. Interestingly, the chemoreflex response exhibited consistency across sexes during both pre-bleo and recovery from ALI (1-mth post-bleo). These findings point to a need for a broader understanding of sex-based variations in lung disorders and underscore the importance of considering sex as a crucial factor in respiratory research. Further exploration of these mechanisms is warranted to advance our knowledge and refine therapeutic strategies tailored to the unique aspects of male and female respiratory physiology.

## Data Availability

The raw data supporting the conclusions of this article will be made available by the authors, without undue reservation.
